# Functional insights into immunoglobulin superfamily proteins in invertebrate neurobiology and immunity

**DOI:** 10.3389/fimmu.2025.1552151

**Published:** 2025-04-02

**Authors:** Hongyu Li, Yijie Zhang, Yunhuan Zhu, Qingzhi Zhao, Jialu Xu, Xianwei Li, Ling Zhao, Hairun Li, Mingcheng Liu, Yuncheng Qian, Xiaofen Zhang, Keda Chen

**Affiliations:** ^1^ Key Laboratory of Artificial Organs and Computational Medicine in Zhejiang Province, Shulan International Medical College, Zhejiang Shuren University, Hangzhou, Zhejiang, China; ^2^ Ocean College, Beibu Gulf University, Qinzhou, Guangxi, China

**Keywords:** IgSF, arthropods, mollusks, nervous system, immune system

## Abstract

The Immunoglobulin Superfamily (IgSF) represents a vital protein family widely distributed in animal genomes, encompassing multifunctional proteins with immunoglobulin-like domains, including immunoglobulins. These proteins play pivotal roles in various biological processes, such as development, differentiation, adhesion, activation, regulation, and signal transduction. While the functions of IgSF in vertebrates are relatively well understood, their roles in invertebrates remain underexplored. This review aims to comprehensively summarize the functions and mechanisms of IgSF in invertebrates, focusing on arthropods, mollusks, and other primitive phyla. In arthropods, research on IgSF has primarily emphasized its roles in the nervous system, especially in axonal and synaptic regulation, and its critical functions in the immune system. Studies in mollusks have predominantly highlighted the immunological functions of IgSF in pathogen recognition, clearance responses, and signal transduction. In contrast, research on protozoa and platyhelminths has mainly focused on identifying IgSF molecules, with relatively limited insights into their functional roles. In sponges, IgSF is primarily associated with cell adhesion and intercellular recognition. By exploring the genetic and protein structural diversity of IgSF in invertebrates, this review reveals their multifunctionality and complexity in biological systems. It not only enhances our understanding of the roles of IgSF in invertebrates but also lays the groundwork for future studies on their potential applications in evolutionary biology and disease models.

## 1 Introduction

Approximately 450 million years ago, the evolution of upper and lower jaws marked a pivotal advancement in early vertebrates, greatly enhancing their hunting efficiency and adaptability to diverse environments. Molecular clock analyses suggest that the origin of gnathostomes can be traced back to the late Ordovician period, underscoring the significance of this evolutionary milestone in vertebrate history (1, 2). The emergence of jawed vertebrates was accompanied by the evolution of a highly developed adaptive immune system (3). This system comprises B lymphocytes and T lymphocytes, which possess the ability to specifically recognize and eliminate pathogens and other harmful substances, serving as the cornerstone of the body’s immune defense. Furthermore, various components of the vertebrate body contribute distinctively to immune protection. For instance, the first line of innate immunity, consisting of physical and chemical barriers such as the skin, mucous membranes, and sweat, plays a critical role in preventing the entry of pathogens and foreign substances (4).

In contrast, invertebrates have existed on Earth far earlier than vertebrates. Their habitats are fraught with threats from diverse pathogenic microorganisms (5). As the oldest and most diverse group of animals on Earth, invertebrates include species that exhibit both the longest lifespans and the simplest anatomical structures. Examples include Porifera and Cnidaria, which are classified as basal metazoans. Despite their comparatively simple physiological organization, the immune systems of invertebrates have demonstrated remarkable efficacy, enabling these organisms to endure and thrive through prolonged evolutionary challenges and competition (6).

From an evolutionary perspective, invertebrates have undergone a transition from simple unicellular organisms to complex multicellular systems. These organisms are classified into multiple phyla, including Protozoa, Placozoa, Porifera, Cnidaria, Platyhelminthes, Nematomorpha, Annelida, Mollusca, Arthropoda, and Echinodermata. Each phylum exhibits distinct biological characteristics and evolutionary trajectories. Throughout their extensive evolutionary history, invertebrates have adapted to diverse ecological environments, encountering a wide variety of complex pathogens. From the deep sea (e.g., Mesozoic sea anemones *Amphianthus inornata* (7), *Gracilechinus affinis* (8), *Neoaulaxinia zingiberadix* (9)) to forests (e.g., Clitellata and Collembola (10)), and from polar to tropical regions, these extreme and variable habitats pose significant challenges to invertebrate immune systems. Traditionally, invertebrates were thought to rely primarily on innate immune systems to defend against pathogenic invasions (11). While jawed vertebrates rely on the immunoglobulin (Ig) family to facilitate adaptive immunity, invertebrates have independently evolved members of the IgSF to adapt to their complex ecological environments. Although the functional mechanisms differ between these groups, this evolutionary development underscores the sophisticated and diverse immune strategies that invertebrates have cultivated to meet their unique ecological challenges (12).

**Figure 1:**
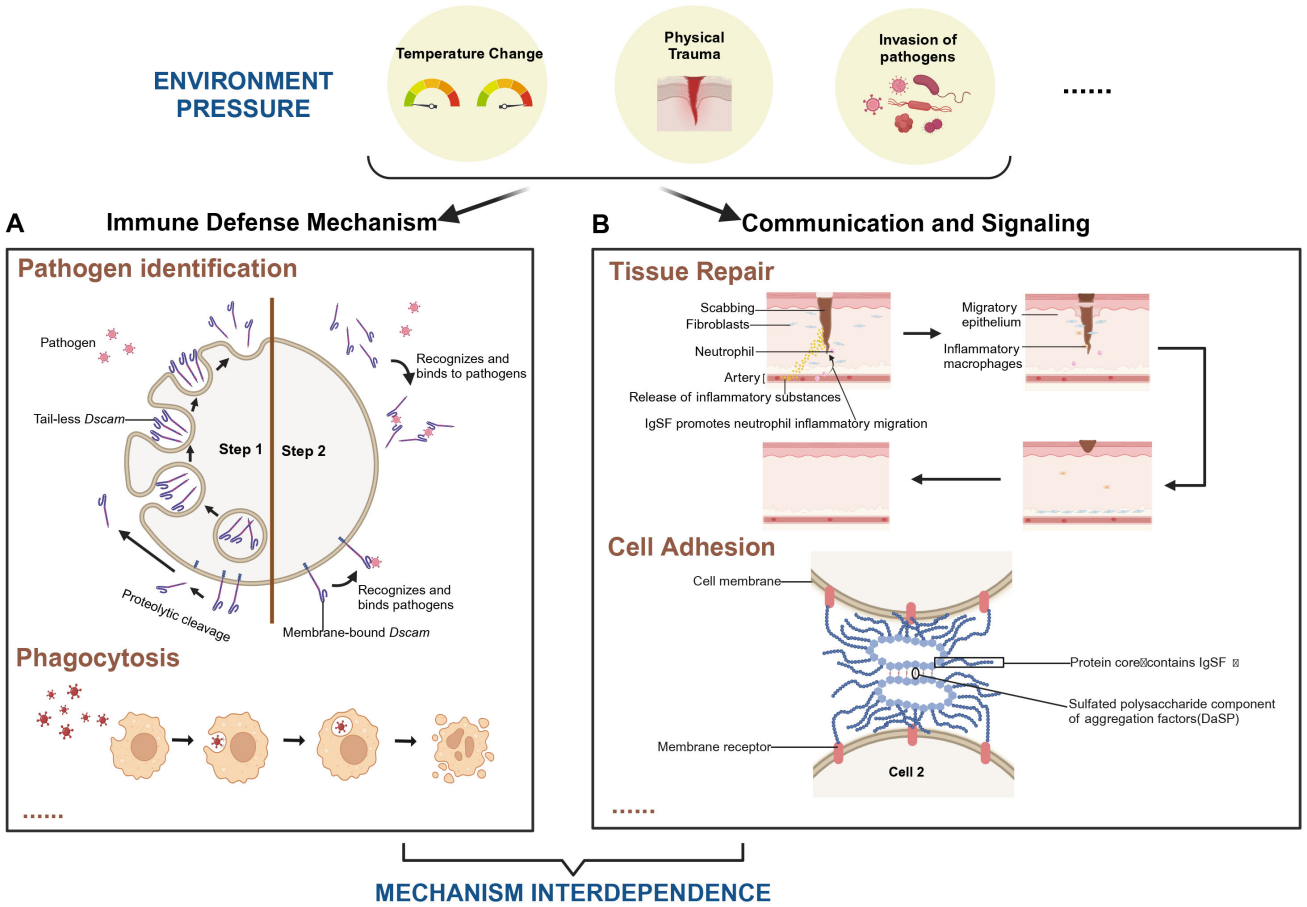
Role of IgSF-mediated immune mechanisms in tissue repair and pathogen clearance under environmental stress. The coordination between immune defense mechanisms and intercellular signaling pathways in supporting tissue repair and pathogen clearance under various environmental stresses, including temperature fluctuations, physical trauma, and pathogen invasion. **(A)** The “Pathogen Identification” module exemplifies the role of *Dscam* in pathogen recognition within arthropods. Step 1: Upon pathogen invasion of arthropod cells, intracellular tail-less *Dscam* is released via exocytosis. Alternatively, proteolytic cleavage of membrane-bound *Dscam* can lead to the shedding of its extracellular domain. Step 2: The released *Dscam* selectively recognizes and binds to specific pathogens, initiating the adhesion process required for subsequent phagocytosis (106). The “Phagocytosis” module illustrates how cells engulf and digest external particles by forming phagosomes. **(B)** The “Tissue Repair” module highlights IgSF’s role in promoting neutrophil migration and initiating inflammation. This accelerates wound healing by attracting fibroblasts and macrophages, which contribute to skin regeneration. The “Cell Adhesion” module focuses on how IgSF-containing proteins, in conjunction with sulfated polysaccharides present in cell aggregation factors, ensure stable cell adhesion, maintaining tissue integrity. Together, these mechanisms underscore the interdependence of immune defense and cellular communicat ion in responding to environmental stresses. They illustrate IgSF’s multifaceted roles in immunity, cell adhesion, and tissue repair, emphasizing its importance in maintaining organismal homeostasis under challenging conditions. Created in https://BioRender.com.

Ig is a specialized class of proteins primarily secreted by B cells in vertebrates, responsible for recognizing and neutralizing foreign antigens such as bacteria and viruses. The IgSF represents a broader family of proteins, encompassing not only immunoglobulins but also numerous other proteins with Ig-like domains (13, 14). Through ligand-receptor interactions, IgSF molecules are involved in diverse biological processes, including development, differentiation, activation, cell adhesion, signal transduction, and immune regulation (15, 16). In **Figure 1**, we depict how IgSF plays a role in pathogen recognition and mediates the clearance of pathogens through phagocytosis under certain external pressures, including changes in temperature, trauma, and pathogen invasion. Additionally, IgSF plays an important role in tissue repair and the processes of intercellular recognition and adhesion.

The Ig-like domain exhibits high diversity while maintaining remarkable conservation. Its characteristic structure consists of a sandwich-like fold composed of two β-sheets, commonly referred to as the Ig fold (17). For example, cell adhesion molecules (CAMs), which are rich in Ig-like domains, play critical roles in cell-cell interactions, particularly between neurons and between neurons and other cell types (18). In the nervous system, IgSF molecules regulate intercellular interactions, contributing to neural development, synapse formation, and the construction and function of neural circuits. The structural diversity and functional properties of IgSF molecules provide key insights into the mechanisms of cell-cell interactions in the nervous system (19).

This paper first explores the diverse characteristics of the IgSF, which are reflected not only in genetic diversity but also in structural diversity trends. Among invertebrates, we focus specifically on two representative groups: arthropods and mollusks. In arthropods, the functions of IgSF are primarily associated with the nervous and immune systems. In the nervous system, IgSF plays a critical role in synaptic signal transmission, while in the immune system, numerous molecules within the IgSF family have been identified as key players in immune responses. In mollusks, the immune functions of IgSF are more pronounced, involving pathogen recognition, immune response activation, and signal reconstruction both inside and outside the cell. Additionally, IgSF plays a significant role in the activation of immune-related genes. Beyond arthropods and mollusks, IgSF also functions in other animal phyla, such as protozoa, plankton, and sponges, where its molecules have been identified and are involved in cell adhesion processes. Our review summarizes current research findings on IgSF in invertebrates, highlighting its diverse functions across different invertebrate phyla, particularly its critical roles in immune defense and intercellular communication. These findings offer new perspectives on the immune mechanisms of these organisms and lay a solid foundation for further studies.

## 2 Immunoglobulin superfamily diversity features

### 2.1 The diversity of immunoglobulin superfamily genes

The IgSF is one of the largest and most diverse protein families in the human body, encompassing over 765 members (15). This diversity is evident not only in the vast number of members but also in the complexity of their gene structures and functional variety.

To illustrate the genetic diversity and the process that generates it, let’s consider the mechanism of Ig gene rearrangement, a critical step in the development of B cells. The gene rearrangement process in both heavy and light chains involves precise joining of variable (V), diversity (D), and joining (J) gene segments through somatic recombination. This process is fundamental to the generation of a vast array of antibodies with different specificities.

During the early stages of B-cell development, V(D)J recombination gradually establishes precursor cells expressing Ig heavy chains (IgH) (20, 21). For instance, in fish, clusters of activation-induced cytidine deaminase-positive (*Aicda+*) cells surrounding “melanomacrophages” have shown evidence of B-cell clonal expansion and frequent somatic mutations in VDJ segments (22). Following gene rearrangement, B cells undergo somatic hypermutation (SHM) and class switch recombination (CSR), enhancing antibody affinity and altering their biological properties while retaining high specificity for antigens (23). In certain species, such as chickens, immune responses are further optimized through immunoglobulin gene conversion (23).

Inter-species genetic diversity also underscores the functional variability of IgSF. For example, in mammals, natural killer cell protein 30 (NKp30) acts as an activating receptor for natural killer cells and features a single exon-encoded VJ-type IgSF domain with a transmembrane region (24). Furthermore, paralogous homologs of the natural cytotoxicity receptor 3 homolog (*NCR3H*) are present in most vertebrates but absent in mammals, suggesting that these gene complexes formed early during whole-genome duplications in jawed vertebrates (24). In humans, Ig gene polymorphisms are also widespread; for instance, the constant heavy chain regions encoded by immunoglobulin heavy chain gamma 1 (*IGHG1*), immunoglobulin heavy chain gamma 2 (*IGHG2*), and immunoglobulin heavy chain gamma 3 (*IGHG3*) exhibit significant individual variation, potentially impacting antibody functions (25).

The mechanisms underlying the diversity of the IgSF share both similarities with and differences from the gene rearrangement processes observed in Ig. As a broad molecular family, IgSF exhibits diverse mechanisms of variation, which differ among its members.

In the adaptive immune system, Ig and T-cell receptors (TCRs) primarily rely on V(D)J recombination to generate diversity, enabling the recognition of a wide range of antigens. For instance, during early B-cell development, V(D)J rearrangement sequentially gives rise to precursor cells expressing the IgH (20, 21). However, not all IgSF members rely on gene rearrangement for diversity. Certain non-immune-related IgSF molecules, such as the Down syndrome cell adhesion molecule (*Dscam*) in arthropods, achieve extensive variability through alternative splicing. This mechanism enables the generation of thousands of distinct isoforms (26), which play crucial roles in both neural development and immune recognition (27).

Beyond genetic mechanisms, the diversity of IgSF members is further shaped by post-translational modifications (PTMs). Modifications such as glycosylation and dephosphorylation (28) can alter protein conformation and function, thereby expanding their biological properties.

Additionally, Gene duplication also plays a significant role in immune diversity among invertebrates. For example, fibrinogen-related proteins (FREPs) in *Biomphalaria glabrata* exhibit high variability through point mutations and recombination (29).

Invertebrates also display IgSF gene diversity. Huene et al. identified 41 members of the *Allorecognition* (*Alr)* gene family within the *Hydractinia symbiolongicarpus* allorecognition complex, highlighting the remarkable diversity of IgSF genes (14). Their unique structural features provide new insights into the evolution of IgSF genes in invertebrates. For another example, *Drosophila* neurons express over 100 different cell adhesion molecule (CAM) genes during development, highlighting the critical role of genetic diversity in nervous system development (30). This extensive genetic diversity forms the foundation for the wide-ranging functions of IgSF, demonstrating its significance in immune systems and other biological processes.

### 2.2 The structural diversity of the immunoglobulin superfamily

The structural diversity of IgSF members is central to their functional versatility (31). This diversity manifests across genomic and domain levels. For instance, in the *Aplysia californica*, two distinct *Aplysia californica* Fibrinogen-related protein (*Ac*FREP) proteins were identified: *Ac*FREP1 lacks introns entirely, whereas *Ac*FREP2 is encoded by four exons (32). Such differences in gene structure directly influence protein structure and function, showcasing IgSF’s remarkable genomic flexibility.

Ig structural diversity arises from complex genetic and molecular mechanisms. This diversity is initially reflected in Ig classification, including five types of heavy chain constant region gene segments (*α*, *β*, *δ*, *μ*, and *ϵ*) and two types of light chain constant region gene segments (*κ* and *λ*), which divide Ig into five classes (IgM, IgD, IgG, IgA, and IgE) and two types (*κ* and *λ*) (33). Moreover, variations in certain amino acid residues in the constant regions of heavy and light chains further lead to subclasses and subtypes (34).

Structurally, greater diversity is derived from variations in the amino acid residues of the variable regions of heavy and light chains, particularly in the complementarity-determining regions (CDRs), with CDR3 being a key site for diversity (33, 35). These changes result in the high diversity of the IgFab fragment, enhancing antibody specificity in antigen binding (36).

Unlike mammalian immunoglobulins, which are classified into five major types based on their function and structure, members of the IgSF in invertebrates exhibit a much broader diversity. Nevertheless, based on their functional and structural characteristics, they can still be categorized into several subfamilies. Below are some typical subfamilies along with representative members.

One of the most prominent subfamilies is the immunoglobulin cell adhesion molecules (IgCAMs). These proteins typically contain multiple Ig-like domains and primarily mediate cell-cell adhesion, playing crucial roles in neural development, synapse formation, and immune response (37). The presence of IgCAMs has been extensively reported across various invertebrate species (30, 38–42). To date, more than 100 IgCAMs have been identified in both vertebrates and invertebrates (43). For instance, N-CAM has been shown to play significant biological roles in *Drosophila* (38) and *Aplysia* (39).

Another key subfamily includes the Ig-like receptors, whose members typically contain Ig-like domains and are involved in immune responses, cell-cell recognition, and pathogen detection. Additionally, the IgSF subfamily associated with Toll-like receptors (TLRs) plays a pivotal role in the immune systems of invertebrates (44). By recognizing pathogen-associated molecular patterns (PAMPs), TLRs initiate immune responses (45). In cnidarians such as *Hydra*, despite the absence of mobile phagocytic cells, immune responses rely predominantly on epithelial cells, which utilize non-conventional TLR signaling pathways (46). Moreover, IgSF members like DSCAM (47–49) share homology with components of the vertebrate adaptive immune system, providing compelling evidence for evolutionary links between immune mechanisms.

Self-recognition molecules represent another crucial subfamily within invertebrate IgSF. For example, in the cnidarian *Hydractinia symbiolongicarpus*, allorecognition proteins such as *Allorecognition 1* (*Alr1*) and *Allorecognition 2* (*Alr2*) play key roles in distinguishing self from non-self (14, 50), highlighting the complexity of invertebrate immune systems. These proteins belong to a 41-gene family within the allorecognition complex and contain unique V-set and I-set Ig domains (14, 50).

Immunoeffector molecules also constitute a significant IgSF subfamily. For instance, the Molluscan Defence Molecule (MDM) identified in *Lymnaea stagnalis* contains multiple Ig-like domains and plays a crucial role in modulating immune defense responses during parasitic infections (51).

Finally, receptor modulatory molecules represent another important subfamily within the invertebrate IgSF. These proteins are involved in fine-tuning receptor-mediated signaling pathways, further expanding the functional diversity of IgSF members in invertebrate immunity.

The IgSF family includes various domain types, such as the V-set, C1-set, C2-set, and I-set, each with unique functional and evolutionary characteristics (31). The core structure consists of four β-strands (b, c, e, and f) forming an antiparallel β-sheet sandwich. This stable core underpins the fundamental functionality of IgSF (52). However, IgSF also exhibits significant structural flexibility. Variations in the topology of edge strands (e.g., a, c1, c2, d, and g) and alterations in inter-sheet angles ensure adaptability across diverse environments (52). Additionally, the high mutation rate of Ig-like domains and variability in disulfide bonds further enhance this flexibility, enabling IgSF to meet various biological demands (53).

The IgSF family encompasses not only immunoglobulins but also numerous critical biomolecules, such as major histocompatibility complex (MHC) molecules, TCRs, myelin protein P0, β2-microglobulin, and Thy-1 protein (54, 55). This member diversity emphasizes the structural and functional complexity of IgSF and underscores its pivotal roles in immune systems and other biological processes.

Furthermore, the various subfamilies of the IgSF not only share structural similarities but also exhibit functional overlap and interconnections. Despite differences in their specific roles, all these families contain Ig-like domains, a common feature that contributes to their morphological and functional resemblance (17). Among the most prominent shared functions across IgSF subfamilies are cell adhesion and signal transduction. IgCAMs exemplify this functional versatility. Proteins such as NCAM and ICAM are crucial for cell adhesion in the nervous and immune systems (38, 39), whereas *Drosophila melanogaster Dscam*, with its alternative splicing domains, guides axon growth and neuronal recognition during neural development. For instance, *Dscam*1 demonstrates remarkable specificity in neuronal differentiation and connectivity, with its splice variants playing distinct roles in neural circuit formation (26). Although *Dscam* exhibits a diverse splicing pattern, it shares functional similarities with IgCAMs in mediating cell adhesion and recognition (56–58), highlighting common mechanisms within the IgSF. However, the precise organizational and assembly mechanisms of *Dscam* at cell adhesion interfaces require further investigation.

## 3 The function of the immunoglobulin superfamily in invertebrates

### 3.1 The role of IgSF in the nervous and immune systems of *Arthropoda*

Arthropods, the most diverse and morphologically varied animal group, play a key role in global ecosystems and were likely early terrestrial and aerial inhabitants. Today, arthropods are widely distributed across nearly all types of ecosystems (59, 60). Within this diverse group, the IgSF not only plays a pivotal role in the nervous system but also contributes significantly to immune responses.

Certain members of the IgSF, such as Fasciclin, Neuroglial, and Amalgam, are found in insects (61–63), indicating that the diversification of IgSF domains began early in the evolution of metazoans. Through gene duplication and subsequent divergence, these domains have evolved into multifunctional units with distinct roles. Fasciclin is renowned for its role in axon guidance and synapse formation, facilitating proper neuronal connections (61). Another CAM, Neuroglian, is crucial for maintaining nervous system integrity and is closely linked to the regulation of neuronal growth and differentiation (41). Amalgam interacts with Fasciclin and Neuroglian, suggesting its synergistic role in establishing and maintaining synaptic connections (62).

#### 3.1.1 IgSF’s impact on synaptic signaling conduction

**Figure 2:**
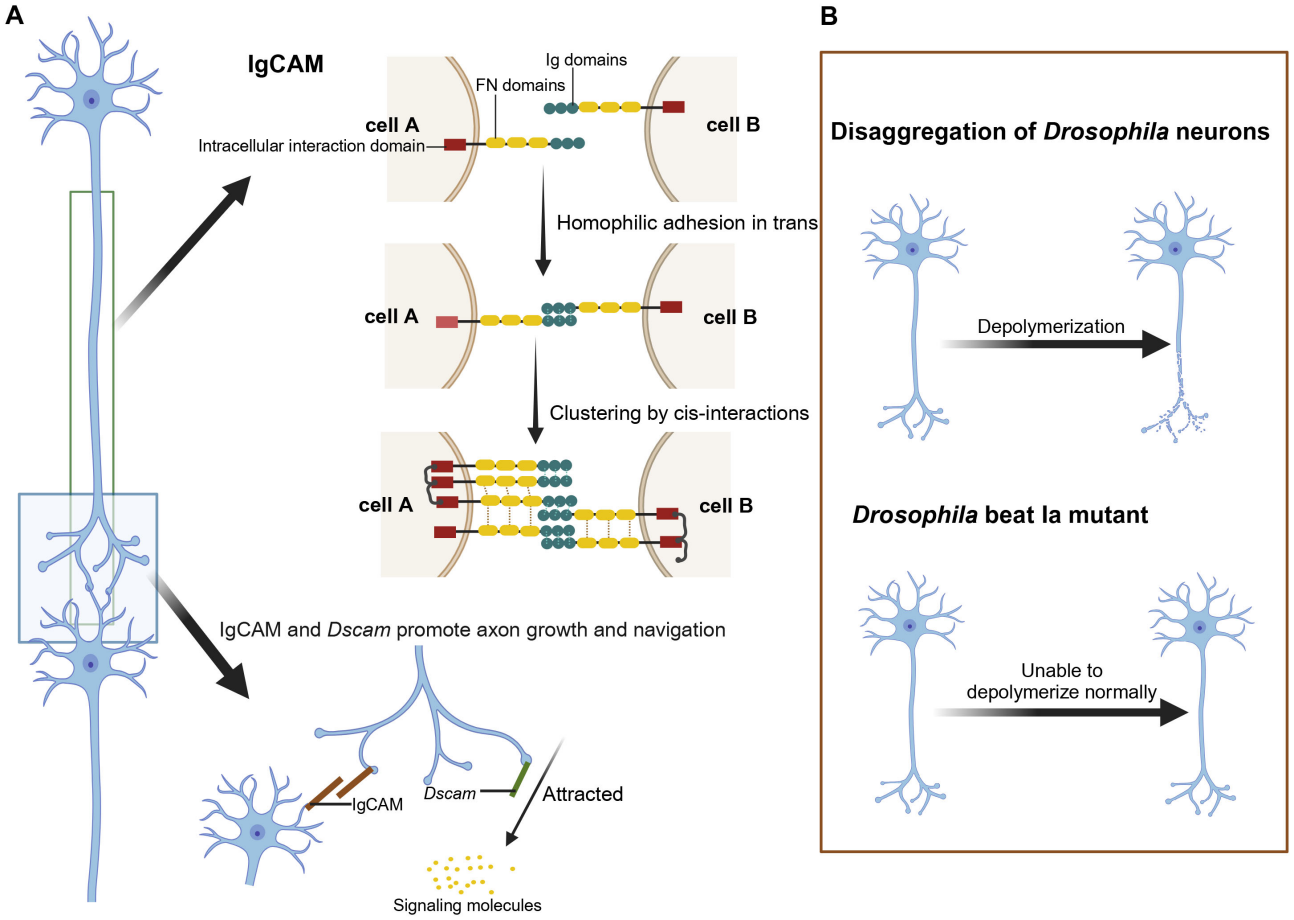
The role of IgCAM and *Dscam* in axon growth and navigation and the effects of their mutations. **(A)** Role of IgCAM and *Dscam* in neuronal connectivity and axon guidance. IgCAM mediates cell-cell adhesion through trans (homoadhesion) and cis interactions, forming stable clusters that bridge cell A and cell B by connecting their Ig and FN domains. Together, IgCAM and *Dscam* support axon extension and directed growth toward signaling molecules. **(B)** Depolymerization in Drosophila neurons: Wild-type neurons undergo controlled depolymerization, while Beat Ia mutants exhibit a failure to properly depolymerize axons. Created in https://BioRender.com.

In the nervous system of *Drosophila*, epithelial IgCAMs play a vital role (37, 42). IgCAMs are involved in axonal growth, fasciculation, neuronal migration, survival, synaptic plasticity, and post-injury regeneration (42). As depicted in **Figure 2A**, IgCAMs interact through their extracellular domains to promote adhesion between axons and guide their growth along neural pathways (64). Notably, at sites of axon-axon interactions, CAMs on growth cones use a specific “zip code” system to ensure precise axonal interactions, forming complex and stable neural network architectures (64).

The *Dscam*, a critical member of the IgSF, is essential for neuronal connectivity in *Drosophila* and some chelicerates (47). Through alternative splicing, *Dscam* generates up to 38,016 isoforms that mediate axonal sorting in the *Drosophila* mushroom body and regulate interactions between adjacent axons (48, 49). In certain arthropods, such as mites and spiders, non-classical forms of *Dscam* proteins lacking typical immunoglobulin and fibronectin III domains have been discovered, potentially having unique functions in neural development (65). Brites et al. defined *Dscam* genes with selective exon arrays as *Dscam*-hv (27). Studies have shown that in *Drosophila*, *Dscam*-hv acts as an axon guidance receptor, ensuring the precise transmission of olfactory receptor neuron signals to specific olfactory regions in the brain (66, 67). Additionally, *Dscam*-hv genes were first identified outside insects in organisms like *Daphnia*, where they contain variable cytoplasmic tail regions. Further research revealed that *Dscam*-hv genes in *Daphnia* are expressed in both brain tissue and hemocytes, with variable expression of exons 4, 6, and 11 reflecting their diverse roles in the nervous and immune systems (27).

Through genetic screening in *Drosophila*, researchers identified the beaten path Ia (beat Ia) gene, the first member characterized among the 14-beat gene families in *Drosophila* (68). The proteins encoded by Beats genes exhibit homology ranging from 24% to 72% in their primary amino acid sequences, primarily concentrated within their Ig domains, with higher homology at the N-terminus that gradually decreases toward the C-terminus. All Beats genes are expressed in the adult *Drosophila* head (68).

Studies indicate that Beat proteins may have anti-adhesive functions (69). In beat Ia mutants, axons display abnormal hyper-adhesion, preventing proper defasciculation, as illustrated in **Figure 2B**, which shows the disaggregation of Drosophila neurons in a beat Ia mutant (68). Conversely, beaten path Ic (beat Ic) promotes axonal bundling, while beat Ia inhibits this process. Their synergistic actions are critical for axon guidance and neural network formation (68).

The IgSF member *Sidekick* (*Sdk*) encodes a large transmembrane protein primarily responsible for neuronal positioning, dynamic cell shape adjustments, and precise axon targeting in the visual system of *Drosophila* (70).

Furthermore, Yu et al. used Mosaic Analysis with a Repressible Cell Marker (MARCM) to screen for cell-autonomous proteins required for axon pruning in *Drosophila* mushroom body (MB) γ neurons (71). They identified a Plexin-D1 like protein with unique domains (Plum), an IgSF transmembrane protein, which is closely associated with intracellular signaling and intercellular interactions during axon pruning. Notably, Plum’s role is confined to axon pruning within MB γ neurons (71).

#### 3.1.2 Role of IgSF in promoting signaling at synapses

IgSF surface proteins play critical roles in synaptic specificity in neuromuscular circuits, visual circuits, and the pathfinding of olfactory neuronal axons (72). Research reveals that *Drosophila* Plexin Receptors (Dprs) and *Drosophila* Interacting Proteins (DIPs) are two major subfamilies of IgSF proteins, offering new perspectives on synaptic specificity regulation (73).

Dprs exhibit specific expression patterns across neuronal clusters and bind to DIP proteins expressed selectively at synapses. The precise expression of specific cell surface proteins (CSPs) is essential for stable and accurate synaptic connections (74). For instance, DIP-α is selectively expressed in two known motor neurons, decisively influencing the correct presynaptic connection of MNISN-1 (73). Synaptic specificity refers to the high precision of neurons in selecting synaptic partners and determining synapse locations (75).

In *Drosophila* leg neuromuscular systems, the transmembrane IgSF proteins DIP-α and Dpr10, located in motor neurons (MN) and target muscles, respectively, are crucial for several developmental stages, including axonal fasciculation, synaptic specificity, and proper synapse formation. Similarly, these proteins participate in synaptic connections within the adult *Drosophila* olfactory and visual systems, as well as larval body-wall neuromuscular junctions (76).

Xu et al. identified a Dpr-ome interaction network comprising 21 Dpr proteins and 11 DIP proteins through an *in vitro* interactome screening. This network exhibits complex interaction patterns (30). During the pupal stage in the optic lobe (OL), neurons expressing specific DIPs often correspond to postsynaptic neurons expressing binding Dprs *in vitro*. Loss of function in DIPs or Dprs results in altered synaptic connections and neuronal death, underscoring their essential roles in synaptic specificity regulation (30).

In *Drosophila* neuromuscular and visual systems, Dpr-DIP interaction networks, determined primarily by shape complementarity, are involved in synaptic connectivity, terminal development, and neuronal survival (77). Single-cell RNA sequencing of embryonic *Drosophila* motor neurons, combined with imaging techniques and genetic perturbation, revealed the importance of homeodomain transcription factors (TFs) and IgSF proteins in synaptic wiring within neuromuscular systems (78).

#### 3.1.3 The role of IgSF in arthropod immune defense

Certain invertebrate proteins with Ig-like domains in the IgSF play crucial roles in host (e.g., insect) immune defenses (79).

Through transcriptomic analysis of *Scylla paramamosain*, researchers identified a novel IgSF member, Leucine-Rich Repeats, and Leucine-rich repeat and immunoglobulin-like domain-containing protein 1 (Lrig-1) (80). This gene is expressed across various tissues and is significantly upregulated in hemocytes during primary and secondary infections with *Vibrio parahaemolyticus* (80). Experiments showed that RNA interference (RNAi) knockdown of Lrig-1 markedly reduced antimicrobial peptide expression, demonstrating its critical role in host immune responses (80).

Another typical IgSF member is Hemolin, encoded by a single gene in the *Hyalophora cecropia*. This gene contains introns located within and between Ig-like domains, exhibiting unique genomic organization. As an immunoglobulin-like protein, Hemolin plays a vital role in insect immune defense by recognizing and binding pathogen surface molecules, thereby promoting pathogen aggregation and clearance (81).

### 3.2 IgSF in immune system functions of mollusks

Mollusks is one of the bilaterally symmetrical eumetazoan phyla, second only to arthropods in biodiversity, with approximately 76,000 extant species currently identified (82–84). This phylum is typically divided into 7 or 8 classes, two of which are entirely extinct. Mollusks exhibit a rich diversity of species and morphological variations, generally characterized by soft bodies protected by hard shells (85). Despite their relatively simple tissue and organ structures, the immune system of mollusks demonstrates a high level of complexity and diversity, especially highlighted in studies of IgSF molecules.

#### 3.2.1 Mollusks use the immune eye IgSF to recognize pathogens

In gastropod mollusks such as *Biomphalaria glabrata*, multiple IgSF proteins have been identified, among which fibrinogen-related protein (*Bg*FREP) plays a crucial role in immune defense. The N-terminal of *Bg*FREP contains two tandem IgSF domains, while its C-terminal features a fibrinogen domain. During parasitic infections, *Bg*FREP levels significantly increase, indicating its pivotal role in immune responses (86, 87). Studies have shown that *Bg*FREP, through its polymorphism and ability to form multimers, binds specifically to parasitic antigens such as *SmPoMucs*, influencing host-parasite compatibility dynamics and serving as a core component of immune defense (88). This mechanism enhances the snail’s ability to recognize and respond to various parasitic antigens, effectively improving its immune defense efficiency.

Additionally, in another freshwater snail, *Lymnaea stagnalis*, an immune-related molecule known as MDM, which contains five C2-like Ig-like domains, was identified. This molecule endows snails with specific immune recognition and regulatory functions and plays a vital role in immune defense during parasitic infections (51). Experiments have also revealed that upon exposure to digenetic trematodes such as *Echinostoma paraensei*, *Biomphalaria glabrata* produces large amounts of hemolymph lectins containing IgSF and fibrinogen domains, aiding in the clearance of invading pathogens (89).

#### 3.2.2 IgSF-mediated immunity to pathogens in mollusks

The functional diversity of *Bg*FREP arises from mechanisms such as exon loss and somatic mutations, enabling it to bind to various parasitic antigens and regulate host-parasite compatibility and resistance (88, 90). Its structure comprises upstream IgSF domains and downstream fibrinogen-related domains (FreD), functioning in pathogen clearance during parasitic infections (91).

Moreover, research indicates that parasitic infections lead to a reduction in MDM levels, thereby weakening the host’s immune recognition capacity. This may serve as a critical strategy for parasites to evade host immune systems (51). In *Sinonovacula constricta*, two fibrinogen-related proteins, *Sc*FREP-1 and *Sc*FREP-2, have been identified. These proteins can recognize and bind to Gram-positive bacteria, Gram-negative bacteria, and pathogen-associated molecular patterns (PAMPs), inducing bacterial agglutination in the presence of calcium ions and ultimately clearing invading pathogens (92). Thus, *Sc*FREP-1 and *Sc*FREP-2 play crucial roles in the innate immunity of clams, offering new perspectives for studying Molluscan immunity (92).

#### 3.2.3 Mollusks transmit intracellular and extracellular immune instructions via IgSF

In *Crassostrea gigas*, a novel IgSF member, *Cg*IgIT2, has been identified (93–95). This protein is predominantly expressed in hemolymph and comprises five extracellular Ig domains, a transmembrane (TM) domain, and a classic immunoreceptor tyrosine-based activation motif (ITAM) (93). *Cg*IgIT2 recognizes various microorganisms in the hemolymph, such as *Vibrio* sp*lendidus* and lipopolysaccharide (LPS), and activates intracellular signal transduction by forming dimers. Specifically, the signaling mechanism involves inducing the production of defensive proteins, such as *Cg*ICP, through the ERK phosphorylation pathway (95).

Further studies reveal that defensive proteins *Cg*CAICP-1, *Cg*ICP-2, and *Cg*LRRIG-1, which contain transmembrane domains, are distributed on the surface of hemocytes. They can recognize and phagocytose *V.* sp*lendidus*, subsequently digesting it within lysosomes. These findings demonstrate that through dimer formation and activation of the ERK phosphorylation signaling pathway, *Cg*IgIT2 plays a vital role in the immune defense of *Crassostrea gigas* (95).

**Figure 3:**
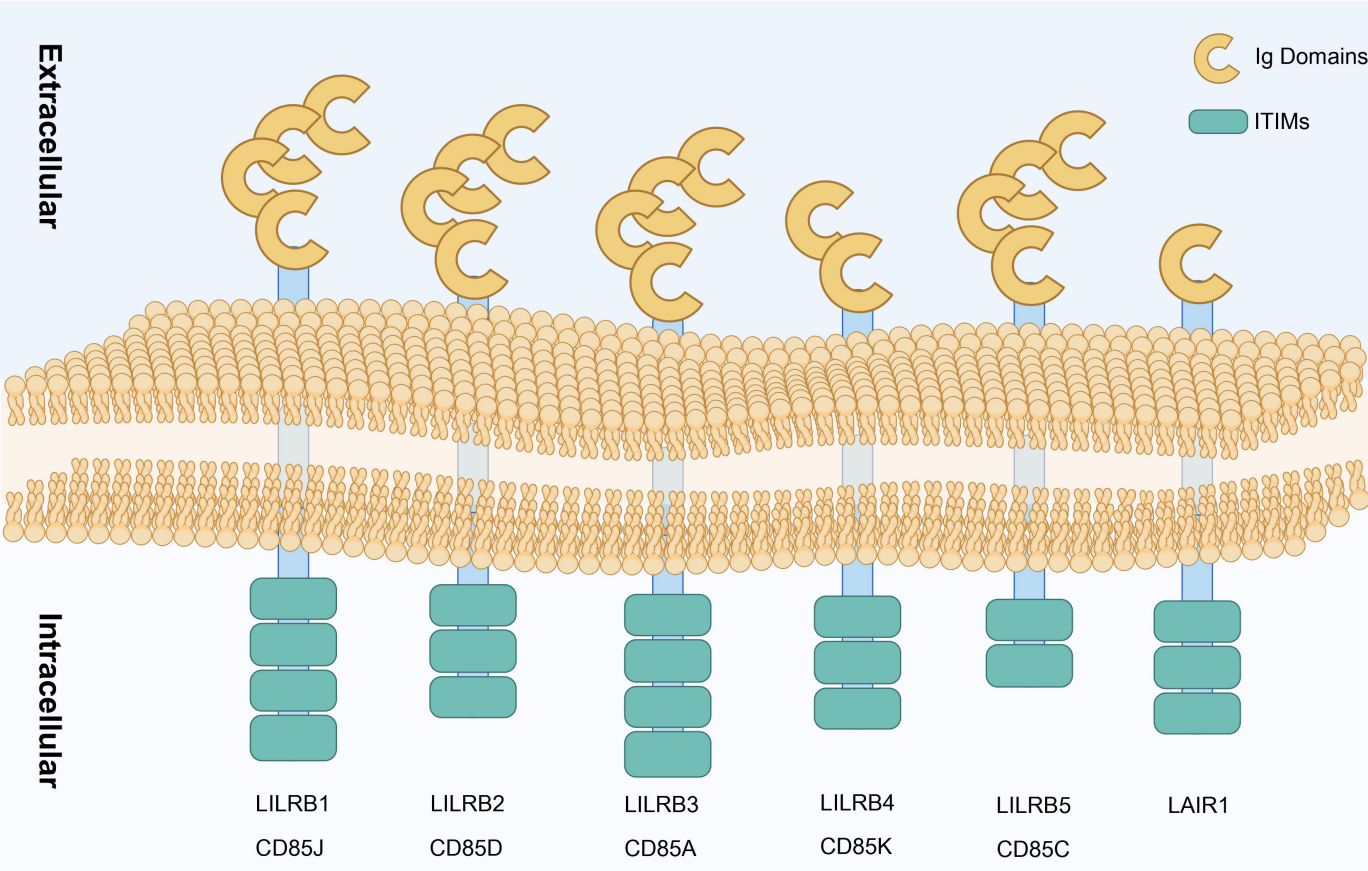
Types of LILRB. Structural representation of LILRB family receptors (LILRB1–LILRB5) and LAIR1 showing their extracellular Ig domains and ITIMs. Each receptor spans the cell membrane, with multiple Ig domains in the extracellular region facilitating ligand binding, while the ITIM in the intracellular region participates in downstream inhibitory signaling, contributing to immune regulation. Created in https://BioRender.com.

Inhibitory leukocyte immunoglobulin-like receptors (LILRBs), a class of single-pass transmembrane glycoproteins (**Figure 3**), were first named in 2001 (63). This family’s extracellular region contains Ig-like domains, while the intracellular region carries immunoreceptor tyrosine-based inhibitory motif (ITIM) (96). The LILRB family includes five members (LILRB1–LILRB5, also known as CD85J, CD85D, CD85A, CD85K, and CD85C) and a related member, LAIR1. As a novel immune checkpoint protein, LILRB’s ITIM structure, similar to CTLA-4 and PD-1, can be directly expressed on the surface of tumor cells, participating in tumor cell growth and survival. This characteristic positions LILRB as a significant target in tumor immunotherapy drug development (97).

#### 3.2.4 IgSF promotes immune gene activation in mollusks

In the *Aplysia californica*, two fibrinogen-related protein genes, *Ac*FREP1 and *Ac*FREP2, have been discovered (32). Although the specific immune responses of *Ac*FREPs have not yet been observed, their homology with the agglutinin functions of *Biomphalaria* suggests that these genes may play similar roles in immune defense (32). However, the functional differences and precise mechanisms of *Ac*FREP1 and *Ac*FREP2 remain subjects for further experimental research.

### 3.3 IgSF in animals of other phyla

#### 3.3.1 Functions of IgSF in protozoa and placozoa

Protozoa are the most primitive and simplest unicellular organisms, often referred to as unicellular animals. Despite their structural simplicity, studies have identified molecules resembling the IgSF in protozoa. For example, receptor tyrosine kinases with Ig-like RTKs have been identified in protozoa (98), suggesting that early invertebrates already possessed fundamental immune and signal transduction systems.

Placozoa represents another evolutionarily significant group of animals. In 1883, the German zoologist F.E. Schulze first described *Trichoplax adhaerens*, a tiny marine organism (99). In 1971, K. Grell adopted the “placula” hypothesis, positioning Placozoa as potential common ancestors of sponges and cnidarians and as the origin of the lineage leading to all higher metazoans (100).

Further studies revealed the presence of a primitive major histocompatibility complex (MHC) in *T. adhaerens*, which is involved in innate immune processes within cells (101). Although no typical markers of adaptive immunity (e.g., MHC class I and II molecules) have been found in Placozoa, these findings provide critical insights into the early evolution of the animal immune system. They highlight the pivotal role of Placozoa in the evolutionary trajectory of immunity and their influence on subsequent higher organisms.

#### 3.3.2 Cellular adhesion functions in sponges

The sponges, also known as Phylum Porifera, represents one of the most basal groups of extant metazoans. Despite their relatively simple body structure and lack of true organ systems, with tissue complexity limited to the cellular level, sponges hold significant importance in the study of invertebrate evolution and biology. For years, sponges have been extensively used as model systems to study cellular adhesion (102, 103).

In sponges, CAMs are surface glycoproteins that play a crucial role in development and tissue stability. These molecules often contain one or more Ig-like repeat sequences (103), which mediate cell-to-cell adhesion and interaction. For example, sponge cell reaggregation depends on an extracellular proteoglycan complex called the aggregation factor. This factor is essential for whole-body regeneration (WBR) through cell reorganization in sponges (104).

Further research has identified glyconectins (GNs), a novel class of proteoglycan-like cell adhesion and recognition molecules, which are widely distributed across various sponges. Researchers Y. Guerardel and G.N. Misevic have highlighted that the functional mechanism of GNs exhibits evolutionary similarities with selective mechanisms in the IgSF, where carbohydrate domains play a crucial role in cell adhesion and recognition (105).

Additionally, in the ancient sponge *Geodia cydonium*, often referred to as a living fossil, researchers J. Kubrycht and J. Borecký identified a specific adhesion molecule, GSMS, which contains Ig-like domain sequences. This discovery provides new insights into the origins of early animals immunity and cell adhesion functions (105).

To provide a comprehensive overview of the roles and distributions of the IgSF members across invertebrates, we have compiled a detailed table (**Table 1**). This table encapsulates the diverse functions of IgSF molecules, ranging from immune defense and neural development to synaptic plasticity and axon guidance.

**Table 1:** The roles and distributions of the IgSF members across invertebrates.

IGSF	Function Description	Types of invertebrates containing this IGSF	References
lrig-1	The immune system participates in the defense against primary and secondary infections with *Vibrio parahaemolyticus* by regulating the expression of antimicrobial peptides.	*Scylla paramamosain*	(80)
Hemolin	Participates in the immune defense response of insects. It can recognize and bind to molecules on the surface of pathogens	*Hyalophora cecropia*	(81)
IgCAM	IgCAM plays a key role by promoting axon growth and fasciculation, supporting neuronal migration and survival, regulating synaptic plasticity, promoting regeneration after trauma, enhancing extracellular domain interactions, and guiding the construction of specific networks.	*Drosophila*	(42) (37) (64)
*Dscam*-hv	As an axon guidance receptor, it helps olfactory receptor neurons accurately transmit signals to the specific area of the brain responsible for smell	*Daphnia*, *Drosophila*, *Chelicerata*	(27) (66) (67)
Beats family	Regulates axon aggregation and disaggregation, which is essential for proper axon guidance and formation of neural networks	*Drosophila*	(68) (69)
*Sidekick* (*Sdk*) gene	Responsible for the positioning of neurons in the visual system and the dynamic adjustment of cell shape and for the positioning of axons	*Drosophila*	(70)
Dpr10	Plays a key role in synaptic selectivity in neuromuscular and visual circuits, and in axonal routing of olfactory neurons	*Drosophila*	(76) (77)
FREPs	Immune recognition and neutralization of toxins, may have immune defense functions homologous to those of Biomphalaria lectins. Recognizes and binds to Gram-positive bacteria, Gram-negative bacteria, and pathogen-associated molecular patterns (PAMPs), and has bacterial agglutination activity	*Biomphalaria glabrata*, *Aplysia californica*, *Sinonovacula constricta*	(20) (32) (76) (86) (88) (89) (90) (91) (92)
MDM (Molluscan Defence Molecule)	Immunomodulatory function, helping mollusks regulate their immune defenses against parasitic infections	*Lymnaea stagnalis*	(51)
*Cg*IgIT2	Recognizes different hemolymph microorganisms, activates intracellular ERK phosphorylation, and promotes the production of defense protein *Cg*ICP	*Crassostrea gigas*	(93) (94) (95)
MHC	Participates in intrinsic immunity within cells	*Trichoplax adhaerens*	(101)
CAM	Cell surface glycoproteins that are prominently expressed during neural development and maturation. CAMs facilitate cell-cell adhesion during development and tissue homeostasis.	*Sponge*	(103)
IG-domain RTK	Receptor tyrosine kinase with immunoglobulin-like domains, involved in immunity and signal transduction	*Protozoa*	(98)
Glyconectins (GNs)	Mediates cell adhesion and recognition, with selectivity mechanisms similar to those in the immunoglobulin superfamily	*Sponge*	(105)

### 3.4 Functional conservation and diversification of the IgSF in nervous and immune systems

The IgSF has maintained remarkable functional conservation throughout evolution, especially in cell recognition, adhesion, and signal transduction. These functions are critical across species, with IgSF-mediated interactions playing key roles in nervous system development and immune defense. In the nervous system, CAMs, a major IgSF subgroup, are essential for neural development. In *Drosophila*, IgSF members such as Fasciclin (61) and Neuroglian (41) regulate axon guidance and synapse formation (41, 61, 62). Similarly, in Porifera, CAMs mediate intercellular adhesion, contributing to tissue stability (103, 104). Despite the conservation of core functions, significant functional diversification has emerged across species. In *Drosophila*, *Dscam* undergoes alternative splicing to produce 38,016 isoforms, allowing precise axon sorting (48, 49). Non-canonical *Dscam* variants in arthropods, like mites and spiders, have distinct neural roles, reflecting functional remodeling for unique neural architectures (65). In the immune system, IgSF diversification has led to more complex regulatory mechanis ms. In mollusks, *Bg*FREP (86–88) and *Sc*FREP (92) not only recognize pathogens but also regulate host-parasite compatibility through polymorphism (86–88, 92). In *Crassostrea gigas*, *Cg*IgIT2 enhances immune defense by activating the ERK phosphorylation pathway (95). Thus, while IgSF retains its core functions in pathogen recognition, its diversification supports species-specific adaptations in both nervous and immune systems, highlighting its versatility.

## 4 Conclusion

This review highlights the remarkable diversity of the IgSF in invertebrates and their pivotal roles in various biological processes. IgSF proteins are integral to key cellular functions such as recognition, adhesion, and signal transduction, with significant involvement in both the immune and nervous systems. Through their structural diversity and specificity, these proteins have laid the foundation for the emergence of adaptive immunity, enabling organisms to efficiently recognize and counteract a wide range of pathogens. Simultaneously, IgSF proteins contribute to critical neurodevelopmental processes, including axon guidance, synapse formation, and the functional maintenance of neural networks, underscoring their essential role in nervous system development.

The review emphasizes the role of IgSF in innate immunity among invertebrates, highlighting how diverse IgSF members utilize molecular recognition mechanisms to detect and neutralize various pathogens in mollusks, demonstrating both specificity and functional complexity. In arthropods, IgSF proteins play crucial roles in neural development, synaptic connectivity, and the precise transmission of neuronal signals, illustrating their multifunctionality across different biological processes.

By comparing the structure and function of IgSF across various organisms, this review traces their evolutionary trajectories, revealing how these proteins have adapted to changing environmental pressures and biological needs. These studies demonstrate that the functional diversification and specialization of IgSF proteins represent a dynamic evolutionary response to external challenges, offering new insights into the complexity of immune and nervous systems.

Advancements in IgSF research will deepen our understanding of their molecular mechanisms in immune defense and neuronal functions, opening the door to potential breakthroughs in disease prevention and neuroscience. Furthermore, studying the evolution of IgSF not only brings insight into their adaptive mechanisms across diverse organisms but also offers innovative solutions in fields such as biomedicine and ecological conservation. These findings enhance our understanding of biological evolution and provide a scientific foundation for developing strategic applications to address environmental challenges and related diseases.
